# Self-reported Recent PrEP Dosing and Drug Detection in an Open Label PrEP Study

**DOI:** 10.1007/s10461-016-1360-7

**Published:** 2016-03-18

**Authors:** K. Rivet Amico, Megha Mehrotra, Vivian I. Avelino-Silva, Vanessa McMahan, Valdilea G. Veloso, Peter Anderson, Juan Guanira, Robert Grant, For the iPrEx Study Team

**Affiliations:** Department of Health Behavior and Health Education, School of Public Health, University of Michigan, Ann Arbor, MI USA; Gladstone Institute of Virology and Immunology, University of California, San Francisco, CA USA; University of Sao Paulo Medical School, Sao Paulo, Brazil; Fundação Oswaldo Cruz, Rio de Janeiro, RJ Brazil; Department of Pharmaceutical Sciences, University of Colorado, Aurora, CO USA; Investigaciones Medicas en Salud, Lima, Peru; University of California, San Francisco, CA USA

**Keywords:** PrEP adherence, Self-report, Drug levels, Open-label, Demonstration project, iPrEx, FTC/TDF

## Abstract

Monitoring adherence to pre-exposure prophylaxis (PrEP) is part of the recommended package for PrEP prescribing, yet ongoing concerns about how to do so confidently are exacerbated by gross discrepancies in reported and actual use in clinical trials. We evaluated concordance between reports of recent PrEP dosing collected via neutral interviewing and drug quantitation in the iPrEx open-label extension, where participants (n = 1172) had the choice to receive or not receive PrEP. Self-report of recent dosing (at least one PrEP dose in the past 3-day) was the most common report (84 % of participants), and among these 83 % did have quantifiable levels of drug. The vast majority of those reporting no doses in the past 3-day (16 % of the sample) did not have quantifiable levels of drug (82 %). Predictors of over-report of dosing included younger age and lower educational attainment. Monitoring recent PrEP use through neutral interviewing may be a productive approach for clinicians to consider in implementation of real-world PrEP. Strategies to capture longer term or prevention-effective PrEP use, particularly for younger cohorts, are needed.

## Introduction

Daily oral HIV pre-exposure prophylaxis (PrEP) with Truvada^®^ (FTC/TDF) has been shown to reduce the risk of HIV infection in several studies [1–5], and is currently approved in the U.S. for HIV prevention [6]. However, as with any medication, its efficacy is highly dependent on drug adherence. In randomized, placebo-controlled trials, adherence estimated by drug levels has varied considerably, with high rates of discrepancy between drug quantitation and participant-dependent adherence measures (e.g., self-report and pill count) [7, 8].

Monitoring PrEP adherence is part of the recommended approach for prescribing PrEP [9], and, given high levels of over-reporting of adherence in PrEP clinical trials, concerns about non-adherence and accurately assessing it may contribute to concerns about offering PrEP [10]. Although testing for drug quantitation is rapidly becoming a “gold standard” for PrEP adherence in research studies, the feasibility of these tests in dried blood spots (DBS), plasma or intracellular assays to real world PrEP roll-out is not certain. In practice, healthcare providers will most likely rely on imperfect patient-dependent measures for monitoring PrEP adherence. Estimates of the overall accuracy of self-reported adherence in the context of placebo-controlled, randomized trials have been mixed. In the randomized controlled phase of iPrEx, self-report measures were fairly concordant with drug quantitation only in US sites, although across sites self-reported non-adherence was largely accurate [11].

Accuracy of self-reported PrEP adherence in open label trials and implementation studies has not yet been characterized in the evidence base, but may differ from placebo-controlled trials in important aspects [8]. Qualitative work from FEM-PrEP where there were dramatic discrepancies between self-report and drug quantitation among women in sub-Saharan Africa identified a number of factors that influenced over-report of product use, including fears of being terminated from the trial [12]. In the context of open label projects and real-world PrEP implementation, termination from a trial is not likely relevant, however desires to please staff and professionals providing PrEP would likely retain relevance. To date, it is unclear if the low accuracy in self-report of study product use will be similarly problematic in open-label demonstration projects. In such projects, participants are explicitly told that discontinuing PrEP at any time is supported and re-starting PrEP after discontinuation is also supported. Thus, there may be less overt pressure to appear persistent or adherent.

In this study, we assessed the accuracy of self-reported recent PrEP use in the context of an open-label trial, where participants had the option to receive or not receive PrEP, using time-matched drug concentrations. Self-reported past 3-days of PrEP dosing was contrasted to measured drug concentrations in participants receiving PrEP in the iPrEx OLE study [13] to determine overall concordance and discrepancies. Factors previously identified as associated with drug concentrations in iPrEx RCT (age, condomless sex) [14] or PrEP uptake (condomless sex) and drug concentrations (age, education, condomless sex, number of partners) in iPrEx OLE [13] were evaluated to identify potential correlates of concordance.

## Methods

### Study Population

iPrEX OLE was an open-label study conducted in 11 diverse sites enrolling participants who were older than 18 years old, male at birth, reported having had anal intercourse with men, and had participated in a randomized trial of oral PrEP (iPrEX [1], ATN 082 [15] or the US Safety study [16]). All participants choosing to receive daily, oral Truvada^®^ PrEP were informed that one at least of their blood specimens collected at some point during their first 12 weeks of receiving PrEP would be evaluated for drug concentrations and results would be shared with them. Of the 1225 participants who chose to receive PrEP during the course of the study, 1172 (96 %) had both a drug concentration assessment and self-reported adherence measure from the first 12 weeks of receiving PrEP, and were included in these analyses.

## Measurement

Self-reported recent adherence to PrEP was collected via neutral interviewing [17] where assurances are specifically provided to participants that the study is interested in accurate reports of adherence, and the interviewer is trained to avoid responding either positively or negatively to the participants’ reports of adherence. The interviewer asked participants to report the dates of the last three doses of PrEP taken. Recent dosing was collected at each study visit for participants who received PrEP at the previous visit. Data was transformed to whether or not the participant had dosed in the previous 3 days. Drug concentrations were determined using blood plasma by liquid chromatography tandom mass spectrometry having a lower limit of quantification of 10 ng/ml, reflecting drug ingestion in the past 2–3 days.

Concordance between self-report of recent dosing was defined as conditions where the participant reported any dose taken in the past 3 days and drug concentrations that were above the lower limit of quantitation (10 ng/ml). Concordance of self-reported non-adherence was identified as report of not having dosed in the past 3 days with below limits of qunatitation (BLQ) drug concentrations. Discordance was defined as having BLQ drug levels at the same time as self-reporting at least 1 day of dosing (specifically, ingestion of one tablet) in the past 3 days. Although we also characterize discordant events where reports of not taking the drug coincided with detectable drug concentrations, this condition has the least applicability because individuals at steady state who stopped ingesting for the 3 days prior to the study visit may have had detectable drug levels and accurately report not having dosed recently. The condition of greatest interest was the most common self-report (having dosed) and its concordance or discordance with time-matched drug quantitation. Correlates of concordance included age (continuous in years), education level (referent less than secondary education, completed secondary education set to 1 and completion of post-secondary education set to 2), and baseline reports of binge drinking (5 or more drinks per drinking occasion in past 30 days; set to 1) and indicators of potential elevated risk for exposure to HIV (report of any condomless receptive anal intercourse and report of one or more HIV-positive sex partners on events-based behavioral survey of sex events over the prior 3-months; dichotomized as 1 vs 0). While evaluation of associations with correlates was exploratory, we hypothesized that stability (higher age, higher education, no binge drinking) and increased risk for HIV exposure (reported condomless anal receptive intercourse or known HIV-positive sex partners) may associate with more accurate reports of use and non-use of PrEP.

### Statistical Analysis

Each of the 4 potential outcomes for self-report of dosing in past 3-day (yes/no) by quantifiable drug concentration (yes/no) were characterized descriptively with positive predictive value (PPV) and negative predictive value (NPV). To determine if over-report of adherence (e.g., reporting dosing when drug levels were BLQ) was associated with demography (age or education), alcohol use, condomless receptive anal intercourse, or having an HIV positive partner at baseline multivariable logistic regression was used. For all analysis, we assumed a two-sided alpha error rate of 0.05. All analyses were performed in Stata version 13.1 (StataCorp. College Station, TX: StataCorp LP).

## Results

In full, 1172 participants contributed concordance/discordance data, and 1101 had complete data for the examination of predictors of drug concentrations in the quantifiable range. Participants were 32 years of age on average (median 29), almost half had some post-secondary education (47 %), 89 % were men who have sex with men and 11 % were transgender women. Few (<10 %) participants reported illicit drug use at baseline. 21 % reported binge drinking at baseline (defined as 5 or more drinks per occasion) (Table 1).

**Table 1:** Characteristics of study participants

	Participants in the analysis of concordance between self-report and drug level N = 1172
Age—years^a^	29 (22–36)
Had post-secondary education, n (%)	539 (47 %)
Transgender identity, n (%)	129 (11 %)
Reported drug use at baseline	117 (10 %)
Reported binge drinking^b^	244 (21 %)

^a^Described as median and interquartile range

^b^Five or more drinks on a typical drinking day in the past month

As indicated in Table 2, the vast majority of participants reported having taken at least one dose in the past 3 days (981/1172, 84 %). The PPV, or percentage of participants who reported drug ingestion in the past 3 days and had quantifiable drug, was 83 % (813/981), 95 % confidence interval (CI) 81.3–84.3 %. Conversely, the probability of over-report was 17 % (168/981), 95 % CI 15.7–18.7 %. Among the 16 % reporting not having taken any PrEP dose in the past 3 days, 82 % had BLQ drug concentrations (156/191; Table 2), 95 % CI of NPV of 81.3–84.3 %. Sensitivity of self-report in predicting detectable concentrations of PrEP was 96 % (95 % CI 94.3–97.1 %); specificity of self-report in predicting drug concentrations below levels of concentration was 48 % (95 % CI 42.6–53.7 %).

**Table 2:** Accuracy of self-reported drug adherence compared with drug quantitation

	Drug concentration ≥10 ng/ml		Total
Self-report:	Yes	No	
One or more doses taken in the past 3 days	813 (83 %)	168 (17 %)	981 (84 %)
No doses taken in the past 3 days	35 (18 %)	156 (82 %)	191 (16 %)
Total	848 (72 %)	324 (28 %)	1172

### Correlates of Drug Quantitation

Among participants with drug quantitation data, 1156 (99 %) could be matched with valid correlates data. Self-reported dosing in past 3 days was highly associated with quantifiable drug in plasma; participants reporting at least one dosing day in the past 3 days had 21.57 times the odds (95 % CI 14.42–32.26) of having drug concentrations ≥10 ng/ml compared to participants reporting no ingestion in the past 3 days in unadjusted logistic regression. This result was independent of study site (logistic regression with a fixed effect for study site, adjusted OR 23.58, 95 % CI 15.35–36.22). Examination of bivariate associations between the set of correlates demonstrated no evidence for multicollinearity.

In the model adjusted for study site, age, education, and alcohol use, condomless receptive anal intercourse, and having an HIV positive partner at baseline, self-reported dosing in the past 3 days persisted as a strong predictor (adjusted OR 22.30, 95 % CI 14.46–34.39, p < 0.0001) of drug quantitation (Table 3). Age was also a statistically significant predictor; for each year increase in age, the odds of having drug in the quantifiable range rose 4 % (95 % CI 2–6 %). Among those reporting recent dosing, age was positively associated with having drug concentrations in the quantifiable range.

**Table 3:** Multivariable predictors of quantifiable drug (n = 1156)

	aOR	95 % CI	p value
Self-reported dosing in the past 3 days	22.30	14.46–34.39	<0.0001
Age (per year increase)	1.04	1.02–1.06	0.001
Post-secondary education	1.22	0.99–1.51	0.06
Baseline binge drinking	0.95	0.65–1.38	0.79
Baseline condomless receptive anal sex	0.92	0.66–1.29	0.63
Baseline one or more HIV-positive partners	0.76	0.44–1.30	0.31

Post-secondary education referent less than secondary education; Baseline binge drinking (yes = 1); Baseline condomless receptive anal sex (yes = 1), Baseline one or more HIV-positive partners (yes = 1)

### Correlates of Over-Reported Drug Adherence

Among participants with concordance data, 1156 (99 %) could be matched with valid correlates data. In a model including age, education, gender identity, condomless receptive anal intercourse and study site, younger age and lower education were associated with over-reported drug adherence (Table 4). For each year decrease in age, the odds of over-reported drug adherence was 1.06 times higher (95 % CI 1.03–1.09, p < 0.001). Compared to participants with post secondary education, participants with less than secondary education had 1.7 times higher odds of over-reporting (95 % CI 1.07–2.75, p = 0.025). Examination of bivariate associations between the set of correlates demonstrated no evidence for multicollinearity.

**Table 4:** Multivariable predictors of over-reported adherence (n = 1156)

	aOR	95 % CI	p value
Age (per year increase)	1.06	1.03–1.08	<0.001
Post-secondary education	1.33	1.08–1.64	0.007
Baseline binge drinking	1.06	0.72–1.56	0.76
Baseline condomless receptive anal sex	1.15	0.81–1.63	0.43
Baseline one or more HIV-positive partners	1.09	0.58–2.07	0.79

Post-secondary education referent less than secondary education; Baseline binge drinking (yes = 1); Baseline condomless receptive anal sex (yes = 1), Baseline one or more HIV-positive partners (yes = 1)

## Discussion

In this study evaluating the concordance of self-reported recent PrEP use and drug quantitation in an open-label PrEP study, we found that self-reported recent PrEP use was a strong predictor of drug quantitation in plasma. The association between self-reported recent PrEP use and drug quantitation was independent of study site; age; education; and alcohol use, condomless receptive anal intercourse, and having an HIV positive partner at baseline. Over-reporting PrEP use was associated with younger age and lower education.

It is likely that asking about PrEP use will be a common approach to identify potential problems with adherence in PrEP rollout and implementation. It is reassuring that responses to simple questions about the last 3 doses taken was indeed highly associated with quantifiable drug in plasma, and clearly reports of not taking drug can be confidently considered indicative of non-use of PrEP.

However, 17 % of those reporting recent dosing in our study had concentrations BLQ which appeared to be associated with lower age and education. Over-reported PrEP use could be driven by a number of variables, including social desirability bias and a differential motivation to participate in the study, where younger individuals with lower years of education may have been more likely to enroll due to secondary benefits of study participation and may have poorly understood or trusted the option to participate without receiving PrEP. Additional efforts to investigate predictors of over-reported PrEP use and to improve accuracy in reporting are warranted.

We are also aware of considerable differences in PrEP use across the different regions in which the research was conducted. Site-specific investigations have suggested that some regions had remarkably high adherence and accuracy in self-report of recent dosing [18]. Site differences in PrEP use more generally likely reflect a constellation of social and economic differences between cohorts of participants. Because we controlled for site effects in our analyses, and did not attempt to construct explanatory models for site differences or site-specific results, our results apply to the full sample and cannot be applied to specific regions, per se.

It is important to note that participants in this study were aware of drug concentrations being tested, although they did not know exactly which sample provided over their first 12-weeks of participation would be evaluated. This may have inflated accuracy of self-report as the specific method to determine drug levels used in the current research is highly sensitive to recent dosing. Thus, white-coat dosing cannot be ruled out. An additional important assumption in our work is that we anticipated that drug would be quantifiable if at least one dose was taken in the prior 3-days at a limit of quantitation of 10 ng/ml. This may have been too stringent a limit for participants whose last dose was at the furthest extreme of “3-days ago.” We examined our data for the presence of such cases and determined only a very small number (6 of 1172; <1 %) reported their last dose 3-days prior to specimen collection and had drug concentrations below the 10 ng/ml limit of quantitation. Thus, it is unlikely that an overly stringent limit of quantitation influenced the current findings.

Our results suggest that self-reported adherence collected via neutral interviewing and focused on highly discrete information (dates of last 3 PrEP doses taken) can be a valuable strategy in assessing adherence. Those reporting not having dosed in the last 3 days in all likelihood are accurate, as are most who report dosing at least once in that time period. In the context of inquiring about recent dosing in a manner that specifically invites participants to report openly, providing assurances for no negative consequences for reported PrEP non-use, self-report was highly predictive of having drug detected. Measurement strategies for assessing adherence to medications via self-report is a robust area of inquiry [19] and increasingly more general estimates of overall adherence are being used over discrete count approaches of doses taken and missed over a specified time period [20]. The current results are specific to an interviewer-collected specific, discrete recall period and may not generalize to alternative methods for collecting self-report dosing information.

In the absence of resources for drug level testing or other more objective measures, self-report with neutral interviewing strategies may be a useful tool for clinicians to check-in on recent dosing. In the more mature area of adherence assessment for antiretroviral therapy (ART) adherence, quality standards have emerged for self-report and other methods of monitoring adherence [19]. Self-report of adherence is a recommended component of clinical care visits [21], and tools for clinical use that support neutral interviewing have been developed [22]. Similar, highly targeted, focused work to identify best practices for monitoring PrEP use in clinical practice, as well as PrEP starts and restarts, and cyclic or periodic PrEP use are needed. Our work suggests potential concordance between neutrally assessed recent dosing and drug concentrations within the quantifiable range. However, recent dosing may not indicate consistent adherence over time or adequate use of PrEP at times of greatest risk for HIV exposure (e.g., prevention-effective adherence [23]). Additional efforts are needed to identify best practices for PrEP monitoring in clinical care with particular focus on novel strategies for younger clients with lower attained education.
